# Iodine in commercial edible iodized salts and assessment of iodine exposure in Sri Lanka

**DOI:** 10.1186/s13690-016-0133-0

**Published:** 2016-05-30

**Authors:** Meththika Vithanage, Indika Herath, S. S. Achinthya, Tharanga Bandara, Lakshika Weerasundara, S. S. Mayakaduwa, Yohan Jayawardhana, Prasanna Kumarathilaka

**Affiliations:** Chemical and Environmental Systems Modeling Research Group, National Institute of Fundamental Studies, Kandy, Sri Lanka

**Keywords:** Hypothyroidism, Iodine Deficiency Disorders (IDD), Iodine Induced Hyperthyroidism (IIH), Universal Salt Iodization (USI), Edible salts, Fortification level, Nutrition, Iodide, Iodate, Risk assessment, Exposure

## Abstract

**Background:**

Iodine is an essential micronutrient used by the thyroid gland in the production of thyroid hormones. Both excessive and insufficient iodine intakes can cause thyroid diseases thus harmful to the human body. Inadequate iodine intake by human body causes Iodine Deficiency Disorders (IDD) and hypothyroidism. Excessive iodine intake causes Iodine Induced Hyperthyroidism (IIH). Universal Salt Iodization (USI) is the most effective way of preventing IDD. This study determined the concentrations of iodine species in commercial edible salt products, the stability of iodine at different conditions and iodine exposure at the consumer level.

**Methods:**

The iodine contents of six commercial edible iodized salts were determined qualitatively and quantitatively for both iodide and iodate. Thereafter, the first three products of highest iodine contents, the stability of iodide at exposed to air and heat was measured after 24 hours. Risk assessment of exposure was done at four levels considering the WHO estimation.

**Results:**

Results revealed that all of the salt products have excess iodine that is above the fortification level of 15–30 mg kg^−1^ level in Sri Lanka. Iodide stability was reduced at the average percentages of 13.1, 10.7 and 11.3. The iodate loss percentages were 0, 5.7 and 0 at open air. The iodide loss percentages at the temperature of 50 °C were 4.6, 7.8 and 8.6 while at 100 °C, loss percentages were 11.1, 11.4 and 15.9 for the same salt products. The iodine exposure at lower consumption during cooking ranged 244.4–432.2 μg/day while 325.9–576.3 μg/day for medium consumption, 407.4–720.4 μg/day for moderate high salt consumptions and 488.8–864.4 μg/day for high salt consumptions. As a total 95.8 % cases can cause IIH and only 4.1 % of them can provide optimal iodine nutrition in a population. Iodine exposure without cooking ranged 305.5–540.3 μg/day for low salt consumption, 407.4–720.4 μg/day for medium consumption and 509.2–900.5 μg/day for moderate high consumption and 611.1–1080.6 μg/day for high salt consumptions.

**Conclusions:**

All of the incidents (100 %) of consumption without cooking at the household level can cause excessive iodine intake and IIH in a population.

## Background

Iodine is an essential trace element for human health [1]. Iodine is used by the thyroid gland in the production of thyroxin (T4) and triiodothyronine (T3) hormones that regulate the metabolism of physiological processes of the human body [1]. However, both excessive and insufficient iodine intakes can cause thyroid diseases thus harmful to the human body [2].

Inadequate iodine intake by human body causes the insufficient synthesis of thyroid hormones, resulting hypothyroidism and Iodine Deficiency Disorders (IDD) [3]. Hypothyroidism is a condition of which the thyroid hormone level in the blood lowers [4] since thyroid glands do not produce enough thyroid hormones [3]. IDD contains a collection of functional and developmental abnormalities such as endemic goiter, abortions, still births, cretinism, retarded physical development, brain damage and irreversible mental retardation [1–3]. Excessive iodine intake from food and water can also have adverse chronic health effect such as Iodine Induced Hyperthyroidism (IIH), thyroiditis, goiter, thyroid papillary cancer and thyrotoxicosis" [5–8]. Long term excessive iodine intake increases the activity of Thyroid Stimulating Hormone (TSH) and reduces thyroid hormone production, through loss of the "escape" phenomenon after the Wolff-Chaikoff effect (inhibits organification in the thyroid gland, the formation of thyroid hormones inside the thyroid follicle, and the release of thyroid hormones into the bloodstream) resulting in low thyroid hormone levels, or hypothyroidism in fetuses and newborns [9]. Chronic exposure of iodine in several milligrams per day (1100 μg/day) can disrupt thyroid function [10]. Correspondingly, a clear relationship between excessive iodine intake and distribution of differentiated thyroid cancer and the risk of IIH after correction of iodine deficiency have been identified [5, 6, 9].

Universal Salt Iodization (USI) is the most effective way of preventing IDD [4]. Salt is an excellent carrier for iodine, as it is consumed at relatively constant, well-defined levels by all individuals within a society, independently of economic status [11]. According to the WHO, the recommended iodine intake for the adults should be 150 μg per day and recommended iodine intake for pregnant women is 200 to 250 μg per day [12]. Hence to provide 150 μg daily requirement of iodine for each person, the salt iodine concentration at the point of production should be 20–40 mg per 1 kg of iodized salt [12]. That recommended level was estimated by WHO, under the assumptions of 20 % loss of iodine from production site to household, 20 % loss when cooking and 10 g of salt as average salt intake per capita [12]. It is understood that the actual availability of iodine in salt depends on its form of fortification, environmental and other factors. On exposure to sunlight, wind, salts iodized as iodide lose a considerable amount of iodine while the salts iodized with iodate has shown no losses. Even when heating, the salts containing potassium iodate retained a high percentage of their original iodine content while the salts iodized with potassium iodide had lost a considerable amount of iodine [13, 14]. The recommended iodine intake is also known as Recommended Dietary Allowances (RDA) that means the average daily level of iodine intake sufficient to meet the nutrient requirements of nearly 97.5 % healthy individuals [15]. According to the WHO, the upper tolerance limit of iodine for adults is 1100 μg/day [2].

The iodination program in Sri Lanka was formally implemented in 1995 to avoid IDD in the population [16]. High prevalence of IDD (30-45 %) has been reported over 20 years from 1995 with an estimation of affecting population as 10 million (Fernando et al., 1989; Fordyce et a., 2003). The causes of IDD prevalency is suggested to be multifactorial, due to the existence of humic substance, clay minerals etc. that exert an influence of the bioavailability of iodine (Dissanayake and Chandrajith, 1996). Hence, drastic reduction of IDD was expected by implementing salt iodination however, still goiter is prominently seen as pockets which could be due to selenium deficiency (Fernando et al., 2015; Fordyce et al., 2000). However at present, it has been reported that there are high prevalence of IIH and slightly high concentrations of iodine in urine especially in female adolescents (213.1 μg/L) in Sri Lanka, which may be due to excessive iodine intake from salt due to lack or regulations and monitoring [17–19]. The iodine fortification level in Sri Lanka is 15–30 mg/kg for a salt packet, according to the Food Regulations (2005). Only a few studies have been conducted on assessing iodine in commercial salt products in Sri Lanka and data are quite old [20]. The authors reported that the iodine content in 68.6 % of the packets was outside the range stipulated by the Sri Lanka Standards Institution (SLSI). In 52.8 %, the mean iodine content was above the recommended upper limit of 40 mg/kg and in 15.8 % below the recommended lower limit of 20 mg/kg. Non-iodised salt is not available in Sri Lankan market today at the same time many salt products coming up to the market and most probably with excessive iodine concentrations. Many comprehensive studies around the world have reported an increase in the IIH followed by the iodisation [21, 22].

Therefore, it is vital to determine their iodine concentrations to check whether they follow the recommended fortification level at the point of production. Since the iodine status is analogous to the iodine exposure, it can be used directly for the risk assessment of iodine exposure which may indirectly provide a prediction about the health condition of the population [23, 24]. Hence, experiments were conducted to assess iodine in commercial edible iodized salts to determine the concentrations of iodine species in commercial edible salt products available in the local market, examine the actual availability of iodine varies with its exposure to air and at different cooking practices and to assess the iodine exposure at consumer level to estimate risks.

## Methods

Six different brands of most commonly sold commercial edible iodized salts in different grocery stores were purchased for the study. The basis for the selection was through a consumer questionnaire and thirty consumers were randomly selected for the questionnaire.

### Qualitative determination of iodine species

The presence of iodide and iodate species in iodized salts were determined according to the procedure given in SLS 79: 1987 [25]. For iodate (IO^−3^) determination, first 2 g of salt was dissolved in 10 ml of distilled water in a test tube. Then 1 ml of 10 % potassium iodide (KI) solution and 2 ml of 1 M sulfuric acid were added. Then few drops of 1 % starch solution was added and checked for the blue coloration [25]. In the case of iodide (I^−^), first 2 g of salt was dissolved in 10 ml of distilled water in a test tube. Then 1 ml of 10 % potassium iodate (KIO_3_) solution and 2 ml of 1 M sulfuric acid were added. Then few drops of 1 % starch solution was added and checked for the blue coloration [25].

### Quantitative determination of iodide in salt 

About five grams of salt was measured accurately and dissolved in 25 ml of distilled water. A 500 μl of Ionic Strength Adjuster (ISA) solution was added, shaken well until it fully dissolves and the iodide concentration was measured by iodide combination electrode. Iodide concentrations of all six salt products were measured in triplicates.

### Quantitative determination of iodate in salt

The iodate contents of iodized salts were measured in triplicates by iodometric titration, according to the procedure given in SLS 79: 1987 (2001) [25]. First, 10 g of salt was dissolved in 100 ml of distilled water then 2 ml of 1 M sulfuric acid, 5 ml of 10 % (m/v) KI solution were added and closed immediately [25]. The mixture was shaken well and kept in dark for 5 minutes. Then the solution was titrated with a standard sodium thiosulfate solution (Na_2_S_2_O_3_) of 0.005 M until a light straw yellow color obtained and then 2 ml of 1 % starch solution were added. The titration was resumed until a colorless end point was obtained. Finally, the total iodine contents of each product were calculated. A blank determination was carried out using 10 g of analytical grade NaCl as a control.

### Variability of iodine when exposed to air

Saturated salt solutions were used to study the variability of iodine when exposed to air. First, the first 03 salt products of highest iodine concentrations were chosen. Then saturated salt solutions were prepared by dissolving 10 g of salt in 25 ml of distilled water. Then all the samples were kept exposed to air inside a laboratory cupboard to mimic the conditions under which salts are usually stored at home for use [14]. The iodide contents of each were measured after 1 hour, 6 hours and 24 hours and iodine loss percentage was calculated [14]. To analyze the variability of iodate, another two sets of samples were prepared in triplicates for each product following the above procedure, one set for measuring initial iodate content and the other set was for iodate content after 48 hours. Iodate contents of both situations were measured by iodometric titrations using 20 ml saturated salt solutions [14, 25].

### Variability of iodine at heating

Three samples of saturated salt solutions for each product were prepared by dissolving 10 g of salt in 25 ml of distilled water. Initial iodide contents of each product were measured in triplicates by iodide combination electrode. Then all the saturated solutions were heated at 50 °C for 2 minutes to mimic mild cooking practice. Then they were kept in a water bath until they reach room temperature. Then the iodide contents were again measured using iodide combination electrode. Another set of samples were prepared in triplicates for each products. The initial iodide content and final iodide content after 100 °C boiling for 2 minutes were measured following the same procedure used for 50 °C.

To analyze the variability of iodate, another two sets of samples were prepared in triplicates for each product following the above procedure. Then the variability of iodate at 50 and 100 °C for 2 minutes was measured using iodometric titration according to the procedure given in SLS 79: 1987 (2001) [25]. For each titration, 20 ml saturated salt solution was used. The loss percentages of iodine in each case were calculated to assess the iodine stability.

### Risk assessment of iodine exposure

Four levels of salt consumptions at low, medium, moderate high and high salt intakes were considered. The average salt intake per capita of population at medium salt consumption was assumed to 10 g/day, according to WHO estimations [12] (Table 1). The low and moderate high intakes were calculated by adding ± 25 % portion of the 10 g salt per capita, to the medium salt consumption which is 10 g/day [12]. High salt consumption was calculated by adding 50 % portion of the 10 g salt per capita, to the medium salt consumption (Table 2). Then iodine exposure and risks were calculated for salt consumptions based on iodine contents of salts, with and without the adjustments for cooking losses which is 20 % [23].

**Table 1:** Salt consumption levels and corresponding salt intake

Consumption level	Salt intake (g/day)
Low	7.5
Medium	10.0
Moderate High	12.5
High	15.0

Source: [4]

**Table 2:** Iodine exposure levels and corresponding iodine status

Exposure level (μg/day)	Iodine status
<30	Severe deficiency
30 – 74	Moderate deficiency
75 – 149	Mild deficiency
150 – 299	Optimal nutrition
300 – 449	Above requirement
>449	Excessive
>1100	Above upper tolerance level

Source: [4, 23]

## Results and Discussion

### Qualitative determination of iodate and iodide species

Qualitative experiments exhibited positive results for iodate, which are blue colorations with the addition of 1 % starch solution. That indicates the presence of iodate (IO^3−^) species in all of the salt products [25]. However, in the case of iodide determination, none of the products gave any color change with the 1 % starch solution. That indicated the absence of iodide species in iodized salt products which has been tested [25]. The iodide (I^−^) ions convert iodate (IO−_3_) to elemental iodine (I_2_). This elemental iodine reacts with the iodide ion (I^−^) to form a tri-iodide anion (I^3−^) and this further reacts with it to give a penta-iodide anion (I^5−^). This penta-iodide anion (I^5−^) forms a visible blue–black complex with starch molecules [20].

### Total iodine content of iodized salts

The results showed that all of the products have exceeded the recommended iodine fortification level of 15–30 mg kg^−1^ of Sri Lanka (Table 3). Although the qualitative methods exhibited no iodide present in the samples, the quantitative experiments indicated high concentrations, which express the less importance of qualitative examinations during standardization tests. The iodide concentrations of saturated salt solutions decreased with the time when exposed to air (Table 4). The loss of iodine as elemental iodine after air oxidation is the reason behind that [14, 26]. The iodine loss percentages of salts were 13.0, 10.7, and 11.2 % for C, F and A products respectively (Table 4). Saturated solutions were not kept beyond 24 hours since the water also can evaporate thus it can cause errors in the results. And in household, the salt solutions are replaced and the new salts crystals are added regularly.

**Table 3:** Total iodine contents of different commercial iodized salts in Sri Lanka, 2015

Product	Iodine as iodate ± SD (mg kg^−1^)	Iodine as iodide ± SD (mg kg^−1^)	Total iodine content ± SD (mg kg^−1^)
A	17.2 ± 1.1	39.4 ± 2.2	56.6 ± 2.5
B	14.3 ± 0.5	38.6 ± 0.4	52.9 ± 0.7
C	31.9 ± 2.4	40.1 ± 2.0	72.0 ± 3.2
D	19.0 ± 0.4	35.4 ± 1.2	54.5 ± 1.3
E	4.2 ± 0.5	36.5 ± 0.9	40.7 ± 1.0
F	18.5 ± 0.0	40.9 ± 0.7	59.4 ± 0.7

A-F are: commercial salt products

**Table 4:** The loss of iodine as iodide and iodate in saturated salt solutions in different conditions

The loss of iodine as iodide in saturated salt solutions at different conditions					
Product	Iodine content ± SD (mg L^−1^)	Final iodine content ± SD (mg L^−1^)	Open Environment ± SD (%)	50 °C ± SD (%)	100 °C ± SD (%)
	Initial	24 h			
C	27.5 ± 2.9	23.9 ± 3.3	13.0 ± 3.5	4.6 ± 1.7	11.1 ± 2.7
F	26.1 ± 0.1	23.3 ± 0.1	10.7 ± 0.2	8.6 ± 0.8	15.9 ± 0.3
A	24.0 ± 1.4	21.3 ± 1.4	11.2 ± 3.6	7.8 ± 2.7	11.4 ± 0.6
The loss of iodine as iodate in saturated salt solutions at different conditions					
C	9.6 ± 0.2	9.6 ± 0.0	0 ± 0.0	1.6 ± 2.8	0 ± 0.0
F	5.7 ± 0.2	5.4 ± 0.2	5.6 ± 0.2	5.5 ± 4.8	0 ± 0.0
A	6.6 ± 0.0	6.6 ± 0.0	0 ± 0.0	0 ± 0.0	0 ± 0.0

According to the results obtained, salt solutions from C and A have not lost their iodine content in the form of iodate after 48 hours at the open environmental conditions (Table 4). Iodate is a stable anion and resistant to get reduced when exposed to the air [26, 27]. However, F product has shown a 5.6 % of loss after 48 hours. That may be due to the presence of hygroscopic impurities like magnesium chloride (MgCl_2_), reducing agents like ferrous ions or lower pH that enhance the reduction of iodate [11, 26, 27]. Factors like impurities, reducing agents, metal ions and pH value vary from one salt product to another. So that, further experiments are required to analyze the activity of those factors that affect the stability of iodine in iodized salts under any environmental condition [26].

### Variability of iodine at heating

According to the results obtained, the average iodine loss increases when the temperature rises [11]. All the solutions were brought back to the room temperature by keeping them in a water bath since the electrode being heat sensitive and high temperatures can cause dysfunction of the electrode (Table 4).

### Risk assessment of iodine exposure

The possible exposure of iodine from each product was calculated under assumptions of 10 g/day as the average salt intake per capita and iodine loss during cooking is 20 % [12, 23] (Table 2). According to the data in the Table 5 with and without the addition by loss during cooking, only 16.6 % of the units at low salt consumptions can cause optimal nutrition (150–299 μg/day) and 83.3 % belong to iodine exposure above requirements (300–449 μg/day). Among medium salt consumption from all salt products, 50 % can cause iodine exposure above requirements while the other 50 % belong to excessive iodine exposure (>449 μg/day). Among moderate high salt consumptions, only 16.6 % of the units show iodine exposure above requirements while the rest of 83.3 % belong to the excessive iodine exposure. High salt consumption from all salt products (100 %) with cooking can cause excessive intake of iodine. Out of those 24 cases including low, medium, moderate high and high salt consumptions, only one of them (4.1 %) can lead to optimal iodine nutrition. And the rest of 95.8 % can possibly cause IIH at a population [4].

**Table 5:** Iodine exposure assessment based on iodine contents of salt, with and without adjustments for cooking losses

Product	Total iodine content ± SD (mg kg^−1^)	Exposure ± SD (μg/day)^a^			
		Low	Medium	Moderate High	High
with the adjustments for cooking losses					
A	56.7 ± 2.5	340.1 ± 13.0	453.5 ± 17.3	566.9 ± 21.6	680.2 ± 26.0
B	52.9 ± 0.7	317.5 ± 1.1	423.4 ± 1.5	529.3 ± 1.9	635.1 ± 2.3
C	72.0 ± 3.2	432.2 ± 25.8	576.3 ± 34.5	720.4 ± 43.1	864.4 ± 51.7
D	54.5 ± 1.3	327.0 ± 6.5	436.0 ± 8.6	545.1 ± 10.8	654.1 ± 12.9
E	40.7 ± 1.0	244.4 ± 2.3	325.9 ± 3.1	407.4 ± 3.9	488.8 ± 4.6
F	59.4 ± 0.7	356.8 ± 4.1	475.8 ± 5.5	594.8 ± 6.9	713.7 ± 8.2
without the adjustments for cooking losses					
A	56.7 ± SD	425.1 ± 16.2	566.9 ± 21.6	708.6 ± 27.0	850.3 ± 32.5
B	52.9 ± SD	396.9 ± 1.4	529.3 ± 1.9	661.6 ± 2.4	793.9 ± 2.9
C	72.0 ± SD	540.3 ± 32.3	720.4 ± 43.1	900.5 ± 53.9	1080.6 ± 64.7
D	54.5 ± SD	408.8 ± 8.1	545.1 ± 10.8	681.3 ± 13.5	817.6 ± 16.2
E	40.7 ± SD	305.5 ± 2.9	407.4 ± 3.9	509.2 ± 4.8	611.1 ± 5.8
F	59.4 ± SD	446.1 ± 5.1	594.8 ± 6.9	743.5 ± 8.6	892.2 ± 10.3

^a^Note: Low, medium, moderate high and high values are correspond to 7.5, 10, 12.5 and 15 g/day salt intake

WHO has estimated the average salt intake as 10 g per capita per day, based on the data throughout the world [4, 12] (Table 1). It was revealed that the salt consumption is higher in Asia than the rest of the world [28]. Central Asia ranks highest in salt consumption and many countries in central Asia consume salt more than 12 g per day [28]. Therefore, the average salt intake per capita in Sri Lanka also can be higher than the estimated value due to same factors stated at above. People in Sri Lanka regularly consume seafood, marine fish, cereals, grains, vegetables, milk, dairy products which are the main food sources of dietary iodine, apart from iodized salts [2]. Processed food made by grains may also contain higher amounts of iodine due to the addition of iodized salt or additives that contain iodine [2].

Geographically, Sri Lanka is an island nearer to the equator. Therefore, the climate is tropical with warm and humid weather conditions year around. Due to warmer and humid climate, the excretion of urine is also high. Therefore, daily average water intake per capita of a population could also be high. Due to those factors, the average salt intake per capita in Sri Lanka also can be higher than the estimated value. But the true value has not been estimated by surveys yet. Therefore, the moderate high (12.5 g/day) and high salt consumption (15 g/day) were considered to mimic the possible average salt intake per capita in Sri Lanka.

In our simulation (Table 5) taking into account the level of salt consumption in the population and the iodine content of the salt brands, it is only in the case of low salt consumption of the brand with the lowest iodine content that excessive iodine intake would not be attained in the population. The susceptible groups are the patients who have autoimmune thyroid disease, thyroiditis or a history of thyroid surgeries [16, 20]. And the rest of 75 % also can cause adverse health effects of IIH and autoimmune thyroid diseases due to the iodine exposure in excessive amounts.

Therefore, almost all of the above cases of salt intake, with or without cooking, at low, medium, and high salt consumptions can cause risks of adverse health effects such as IIH [2]. However, most of people in a population are tolerable to the excessive iodine intake from food, water and other supplements like drugs [2]. Only the susceptible individuals are the ones at the risk of IIH and the actual risks for each individual are influenced by many variables including age, gender, genetic predisposition, environmental factors, personal history of thyroid diseases, concurring diseases, and some medications [9]. Tolerable upper level (UL) for iodine is 1100 μg/day [2].

The potential adverse effects of iodine intake above the tolerable UL include dysfunction of thyroid gland, thyroiditis, goiter, hyperthyroidism, sensitivity reactions, thyroid papillary cancers and acute responses [2]. However, those effects are related with the chronic exposure of excessive iodine [4]. Acute exposure of iodine can be occurred after sudden intake of doses of many grams at once [2]. Effects of acute excessive iodine exposure are mouth and stomach burnings, abdominal pain, nausea, vomiting, diarrhea, weak pulse, cardiac irritability and coma [2]. However, studies on time durations for the acute exposure have not been performed yet. Hence, further studies are required in future to assess the effects of acute iodine exposure on humans most probably using lab rats [16]. There is a need for proper monitoring of the salt iodization programs to achieve an acceptable and optimal iodine status in the population. To achieve it, rigorous measures should be implemented to ensure that manufacturers of iodized salt are committed to the recommended specifications.

## Conclusions

All commercial iodized salt products that were collected from the local market contained iodine in both forms of iodide and iodate. All products contained iodine in excess of the recommended level 15 – 30 mg/kg. As consequence, there is a need for proper monitoring of the salt iodization island-wide to achieve an acceptable and optimal iodine status in the population. Chronic exposure to high iodine concentrations is worrying in view of possible iodine induced immune phenomena. Daily average iodine intake per capita and iodine loss during domestic cooking were assumed to be 10 g salt/day and 20 % loss respectively. Based on the risk assessment, it appears that the iodine consumption among the Sri Lankan population may be causing excessive exposure to iodine. The incidences of IIH among the public may have to be investigated more thoroughly. Depending on the outcome, the health authorities may want to recommend appropriate level of salt addition during cooking so that long term health effect can be mitigated or avoided. It may be prudent to make both iodised and non-iodised salt available to the public, keeping the price of both forms the same so that the general population may choose. The adequate supervision of salt iodination should be complemented by periodical tests carry out by the SLSI for the quality of salt, both locally produced and imported, and the packing material should follow standards to minimise losses during transport and storage. That may reduce the risk by excessive iodine fortification of salt which is essential to control IDD. More efforts on educating the general public and strengthen their knowledge would be beneficial in order to prevent excessive salt usage and IIH. Further studies are required to assess the health effects of chronic iodine exposure where one may consume more than the UL for iodine, 1100 μg/day.
